# Gender differences in oral appliance treatment of obstructive sleep apnea

**DOI:** 10.1007/s11325-024-03019-y

**Published:** 2024-05-21

**Authors:** Anette Fransson, Eva Nohlert, Åke Tegelberg, Göran Isacsson

**Affiliations:** 1 Department of Research, Örebro University Hospital, Region Örebro County and Faculty of Medicine Health, Örebro University, Örebro, Sweden; 2 Region Västmanland – Uppsala University, Centre for Clinical Research, Västmanland Hospital, Västerås, Sweden; 3 Department of Orofacial pain and jaw function, Malmö University, Malmö, Sweden; 4 Department of Orofacial Pain and jaw function, Västmanland Hospital, Västerås, Sweden

**Keywords:** Gender, Mandibular advancement device, Obstructive sleep apnea, Oral appliance, Randomized clinical trial, Treatment

## Abstract

**Purpose:**

Although overall success rates for treating obstructive sleep apnea (OSA) with an oral appliance (OA) are high, they are significantly higher among females. To verify published data, the study’s purpose was to evaluate a participant sample after one year of OA use. The primary outcome was treatment response, with responders defined as having an apnea-hypopnea index (AHI) < 10 at follow-up and/or reduced by ≥50% of baseline. Secondary measures were from standardized questionnaires.

**Methods:**

A sample of 314 participants, predominately with moderate-to-severe OSA, were enrolled and instructed to use an OA every night. At baseline and one-year follow-up, polygraphic recordings and questionnaires, including sleepiness (measured using the Epworth sleepiness scale) and quality-of-life (measured using the Functional Outcomes of Sleep Questionnaire), were collected.

**Results:**

Among the 314 participants, 192 completed the one-year evaluation: 51 females (27%) and 141 males (73%). Overall, OA treatment resulted in 78% and 77% responders among females and males, respectively. Neither the difference in improvement nor the absolute change in AHI differed significantly based on gender, at any OSA severity level. There were no significant gender differences in sleepiness or quality of life. Treatment-related adverse reactions were more common among females.

**Conclusion:**

Both females and males with OSA respond well to OA therapy, with nonsignificant gender differences in outcomes. Thus, the hypothesis that females respond better to OA treatment is rejected.

## Introduction

Oral appliance (OA) therapy is well-established and frequently used to treat patients with obstructive sleep apnea (OSA). OA therapy is specifically recommended for patients with mild-to-moderate OSA, and those with a severe condition experiencing adverse effects from, or not tolerating, positive airway pressure (PAP) therapy, [1–3]. OAs are available in different designs and are individually adjusted to push the lower jaw forward. The OA holds the lower jaw in a protruded advanced position, preventing upper airway collapse during sleep and thus facilitating continuous respiration [2].

Overall, OSA prevalence is lower in females than in males, in addition to gender differences in disease manifestation and presentation [4]. Though the mechanisms underlying gender differences in OSA prevalence are not fully understood, contributing factors may include obesity, upper airway anatomy, breathing control, hormones, and aging. Compared with females, males have a longer collapsible oropharynx and their posterior tongue is larger and fatter [5].

Several factors have been associated with favorable OA treatment outcomes. Less severe OSA, younger age, lower body mass index (BMI), smaller neck circumference, and female gender are all indicators of treatment success [6]. In a relatively large study of 1,084 participants, Nigro et al [7] reported that females with OSA are older, have less severe OSA, and are more obese than males with OSA. They also reported that females have more nonspecific OSA symptoms such as tiredness, insomnia, morning headache, restless legs syndrome, and higher Epworth sleepiness scale (ESS) scores.

Vecchierini et al [8] concluded that the overall treatment success rate, defined as a 50% apnea-hypopnea index (AHI) reduction, is significantly higher in females than in males (89% and 76%, respectively) following OA therapy. In their group with severe OSA, the gender difference was even greater—100% and 68%, respectively. That study confirmed earlier findings that neck circumference is a significant predictor of OA treatment success in females, who usually have a smaller neck [9]. These gender differences decrease with age [10]. In a meta-analysis Chen et al [11] also found a significantly higher rate of responders to treatment, odds ratio 0.71, among females compared to males.

Research on OA treatment and gender differences has usually focused on objective polysomnographic measures, and less on patient experience with the intervention [12]. Thus, the study’s purpose was to evaluate gender effects, on both objectively and subjectively experienced treatment outcomes, after one year of OA therapy to treat predominately moderate-to-severe OSA. The study hypothesis was that females with OSA respond to OA treatment better than males on objective response rate and subjective experience.

## Methods

### Study design

**Fig. 1:**
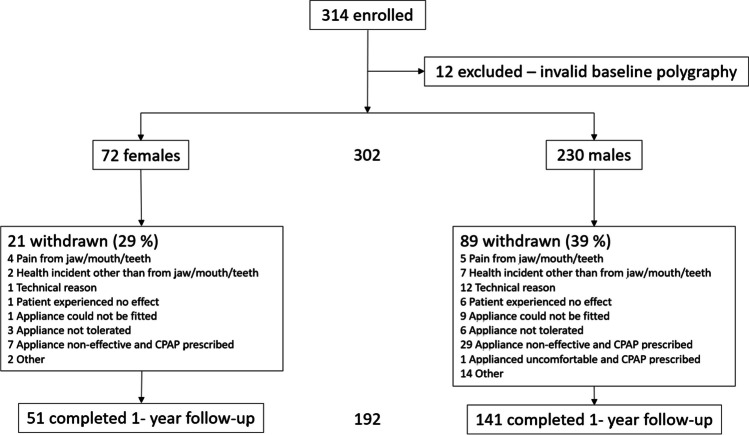
Study flowchart related to gender at baseline and one-year treatment follow-up

This was a secondary analysis of a multicenter, prospective trial focused primarily on the treatment effects of two OA modalities (i.e., monobloc and bibloc). The larger study results showed equivalent efficacy for OA type [13, 14], thus the sample was combined to analyze gender differences herein. The larger study design and OA details have been described elsewhere [13, 14]. Briefly, of the 314 enrolled participants, 12 were excluded for invalid baseline polysomnography. Thus, a sample of 302 participants with OSA (Fig. 1) comprised the current sample for gender-based treatment effect analyses.

The study was conducted in accordance with the principles of the Declaration of Helsinki and Good Clinical Practice. The Uppsala, Sweden Regional Ethical Review Board approved the study on February 19, 2014 (#2014/021). The trial is registered on ClinicalTrials.gov (NCT02148510).

Each participant visited the clinic at least five times. A single-night at-home baseline polygraphy was performed just before the start of treatment. Additional polygraphy were done with concomitant OA use after eight treatment weeks (for interim analysis) and at the end of the first treatment year. Subjective questionnaires were completed at both baseline and one-year follow-up.

### Study sample

Consecutive eligible participants with a verified OSA diagnosis, with AHI ≥ 15 (according to the referral), were referred by physicians for OA treatment. The inclusion criteria were enough teeth to enable OA retention, at least one molar in each quadrant, a maximum mandibular protrusion capacity of ≥6 mm, providing written informed consent, understanding instructions for the portable polygraphy equipment, being able to communicate in Swedish, and valid baseline and one-year polygraphies. The exclusion criteria were age < 18 years, BMI > 35 kg/m^2^, use of PAP therapy or an OA in the past month, functional jaw problem treated within the past year, jaw pain or locking at the baseline examination, inability to follow the study instructions (as judged by the investigator), and hypersensitivity to any of the appliance components.

### Polygraphic recordings

At baseline, all participants underwent a single night of at-home polygraphy (NOX-T3®; Nox Medical, Reykjavik, Iceland) without any respiratory support. At the one-year evaluation, polygraphy was repeated with concomitant OA use. Noxturnal™ software (Nox Medical v. 3.3.0-6715) was used for full-night analysis of each participant’s polygraphy data. Analysis settings were as follows: apnea was scored at a 90% drop in airflow signal for 10–120 seconds and hypopnea was scored at a 30% drop in the flow signal for 10–120 seconds, followed by a 3% drop in SpO_2_. Blinded polygraphy interpretation was made by two experienced hospital technicians at the Västmanland County Hospital Physiology Unit. A minimum of four interpretable hours of sleep was required; if this was impossible, polygraphy was repeated on another night. An interim polygram was collected after about eight OA treatment weeks. Those with unfavorable AHI reductions received further mandibular advancement (i.e., titration). If additional advancement was impossible, the participant was offered PAP therapy and withdrawn from the study.

### Oral appliance and adjustments

Monobloc and bibloc OAs were used, as previously described by Tegelberg et al (13). OAs were constructed with the mandibular advancement degree determined using the George Gauge^TM^ instrument for bite construction [15], set to mandibular advancement to 75% maximum protrusion capacity, or at least 5 mm advancement. At the first checkup visit after the second treatment week, the patient was asked to report the subjective effects; if a partner was present, their evaluation of the treatment effect was also solicited. If these responses were not satisfactory, the OA was adjusted. This was mostly done to advance the bite but could also be retruded due to discomfort. No regular titration procedure was done, but the OA was adjusted if the patient was dissatisfied.

### Outcomes

The primary outcome measure was the treatment response rate at the one-year follow-up, on which females and males were compared. The definition of treatment response was AHI < 10 at follow-up and/or a ≥50% reduction from baseline AHI.

Several secondary outcome variables were also analyzed, including a) the Patient Global Impression of Change (PGIC), a 7-point scale (from “very much improved” to “very much worse”) on which the participant rated any change in their overall status since beginning treatment [16]; b) the Swedish-validated Functional Outcomes of Sleep Questionnaire (FOSQ), a standardized evaluation of sleepiness and activities of daily living [17]; c) the ESS [18], an 11-point Likert scale (0 = no sleepiness; 10 = worst imaginable sleepiness) with response to the statement “Grade your inconvenience of sleepiness in the morning and during the day”; and d) the occurrence of headache, once a week or more, during the past month. In addition to AHI, several polygraphic and clinical measures were also analyzed.

Treatment compliance was evaluated by asking the participant to record the number of nights, and during what proportion of sleeping time on those nights, the appliance was used during the week prior to the one-year evaluation. Treatment-related adverse reactions, spontaneously reported and investigator-observed, were registered throughout the study period. The investigators judged adverse reactions and their relations to the study treatment as probably, possibly, or unlikely.

### Statistical analysis

The primary study objective was to evaluate respiratory efficacy in terms of response rate after one year of OA therapy. Primary efficacy analysis was made on the per-protocol population. Gender differences were analyzed for categorical variables using Pearson’s chi-square tests and for continuous variables using Student’s *t* tests. The McNemar change test was used to analyze change over time in headache (categorical variable). A *p* < .05 was considered statistically significant. IBM SPSS, version 26 (IBM Corp, Armonk, NY, USA) was used for statistical analyses.

## Results

Out of 314 enrolled participants, 192 completed the one-year evaluation. Among these, 51 (27%) were female and 141 (73%) were male. The proportions of female and male participants who discontinued the study were 29% and 39%, respectively. The most common reason for discontinuation was ineffective OA therapy and subsequent prescription of PAP therapy. The study flow chart is shown in Fig. 1. The mean durations for one-year follow-up were 12.4 months (SD 2.5) for females and 12.5 months (SD 1.0) for males. Baseline characteristics and gender differences in the per-protocol population are described in Table 1. With the exceptions of significant differences in the absolute range of mandibular protrusion and participant age, no other significant gender differences were found.

**Table 1:** Participant demographic characteristics at baseline; per-protocol population

	Females (*n* = 51)	Males (*n* = 141)	*p*
Age, years	60.5 (8.5)	53.2 (11.2)	<.001
BMI, kg/m^2^	27.3 (3.8)	27.9 (3.1)	.219
AHI overall, events/h	23.2 (13.8)	24.4 (13.1)	.576
Mild OSA (AHI ≥ 5 to < 15), n (%)	17 (33)	37 (26)	.585
Moderate OSA (AHI 15–29), n (%)	19 (37)	62 (44)	
Severe OSA (AHI ≥30), n (%)	15 (29)	42 (30)	
ODI, events/h	21.5 (13.1)	23.2 (12.8)	.414
Average SpO_2_, (%)	93.2 (1.6)	92.8 (1.6)	.119
SpO_2_ time <90% (% of sleep time)	6.4 (13.8)	8.7 (14.9)	.332
*ESS*	9.3 (5.3)	9.5 (4.7)	.817
Maximum mandibular range of motion*, mm	10.8 (2.0)	12.1 (2.4)	.001

Data are mean (SD) or number of participants (%)

*AHI* apnea-hypopnea index: *OSA* obstructive sleep apnea: *ODI* oxygen desaturation index: *SpO*_*2*_ arterial oxygen saturation: *ESS* Epworth sleepiness scale* Mandibular advancement measured using the George Gauge instrument

*P*-values based on Student’s *t* test or Pearson’s chi-square test

Including all OSA severity groups, OA treatment resulted in 78% female and 77% male responders (Table 2). Analyzing gender differences based on OA severity groups also revealed nonsignificant gender differences in responder proportions. Nor were there gender differences in improvement, including absolute change in AHI, oxygen desaturation index, or sleep time when SpO_2_ < 90%, regardless of OSA severity. However, the change in average SpO_2_ was significantly higher in females (*p* = 0.022) (Table 3).

**Table 2:** The proportion of female and male responders after one-year of treatment with an oral appliance; per-protocol analysis (females total n=51; males total n=141)

	Females (*n* = 51)	Males (*n* = 141)	*p*
All AHI severities	40 (78)	109 (77)	.869
Mild (baseline AHI < 15)	15 (37)	29 (27)	.431
Moderate (baseline AHI = 15–29)	14 (35)	46 (42)	
Severe (baseline AHI ≥ 30)	11 (27)	34 (31)	

Data are the number of participants (%)

*AHI* apnea-hypopnea index: OSA, obstructive sleep apnea

*P*-values tested by Pearson’s chi-square test

Responder definition: evaluation visit AHI < 10 and/or ≥50% reduction of baseline AHI

**Table 3:** The absolute change in polygraphy variables from baseline without treatment to evaluation with an oral appliance in place at one-year related to gender. Per-protocol analysis

	Females		Males		Females vs Males	
	n	Difference mean (SD)	n	Difference mean (SD)	Difference between group means (95% CI)	*p*
All AHI severities	51	–14.2 (11.8)	141	–14.0 (15.5)	0.2 (–4.5 – 5.0)	.918
Mild (baseline AHI < 15)	17	–3.6 (6.0)	37	–1.4 (7.1)	2.2 (–1.8 – 6.2)	.281
Moderate (baseline AHI = 15–29)	19	–14.1 (8.4)	62	–11.5 (13.1)	2.6 (–3.8 – 9.0)	.420
Severe (baseline AHI ≥ 30)	15	–26.5 (8.1)	42	–28.8 (12.1)	–2.3 (–9.0 – 4.5)	.506
ODI	51	–13.2 (11.6)	141	–13.2 (14.5)	0.01 (–4.5 – 4.5)	.997
Average SpO_2_ (%)	51	–0.4 (1.1)	141	–0.01 (1.2)	0.4 (0.1 – 0.8)	.022
SpO_2_ time <90% (% of sleep time)	51	0.4 (8.4)	141	–2.6 (17.4)	–3.0 (–8.0 – 2.0)	.238

*OSA* obstructive sleep apnea: *AHI* apnea-hypopnea index: *ODI* oxygen desaturation index: *SpO*_*2*_ arterial oxygen saturation: *CI* confidence interval

*p*-values from Student’s *t*-test

Participant self-reported adherence to using the OA in the week before the one-year follow-up was a mean of 6.6 (SD 1.0) nights for females and 6.1 (SD 1.6) nights for males. The mean proportion of sleep time during which the OA was used per night during the week before follow-up was 90% (SD 20%) in females and 88% (SD 21%) in males.

Improvement in excessive daytime sleepiness estimated using the ESS did not differ significantly between females (4.2 points) and males (3.6 points). Nor did the Likert scale for rating sleepiness in the morning and during the daytime differ significantly between the genders (Table 4).

**Table 4:** Absolute change in Epworth sleepiness scale and Functional Outcomes of Sleep Questionnaire from baseline to one-year follow-up, by gender; per-protocol analysis

	Females		Males		Females vs Males	
	n	Difference mean (SD)	n	Difference mean (SD)	Difference between groups mean (95% CI)	*p*
ESS	51	–4.2 (4.2)	141	–3.6 (3.8)	0.6 (–0.7 – 1.8)	.388
Sleepiness in the morning*	51	–1.9 (2.5)	141	–1.6 (2.4)	0.3 (–0.5 – 1.0)	.500
Sleepiness during the day*	51	–2.0 (2.4)	141	–1.7 (2.0)	0.4 (–0.3 – 1.0)	.271
FOSQ total	23	1.1 (1.4)	92	1.1 (1.4)	0.01 (–0.6 – 0.7)	.977

*ESS* Epworth sleepiness scale: *SD* standard deviation: *FOSQ* Functional Outcomes of Sleep Questionnaire

*In response to the statement: “Grade your inconvenience of sleepiness in the morning respectively during the day by circling the number (Likert scale 0 = no sleepiness, 10 = worst sleepiness imaginable) that best describes the mean for the past week.”

*p*-values from Student’s *t* test

Results from the FOSQ also showed a nonsignificant gender difference (Table 4).

**Fig. 2:**
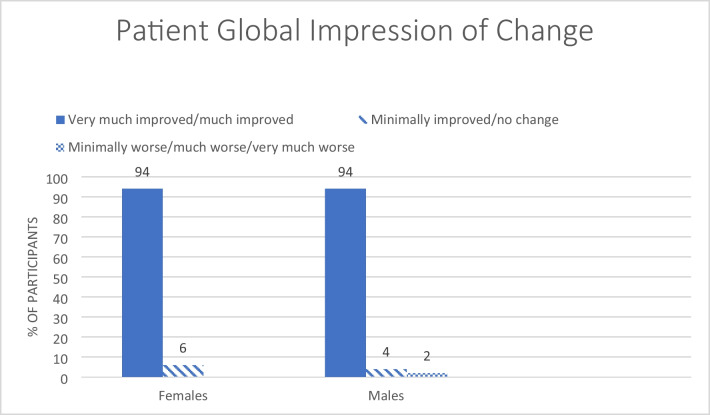
Patient global impression of change, a 7-point scale, with participant rating of the overall treatment effect at one-year follow-up. Chi-square analysis (*p* = .353)

Likewise, 94% of both females and males scored their overall treatment experience status on the PGIC as “very much or much improved” (Fig. 2).

Headache frequency of at least once a week decreased significantly from a mean of 71% to 57% among females (*p* = .012) and from 67% to 47% in males (*p* = .035) but did not show a significant gender difference.

There were more treatment-related adverse reactions among females, with 37% experiencing one or more events, compared with 29% of males (Table 5). Unspecified complaints regarding the mouth or jaws were most common, followed by complaints about the teeth and temporomandibular joints. No serious adverse reactions were reported or observed.

**Table 5:** Incidence of reported and observed treatment-related adverse reactions from start of treatment to one-year follow-up; intention-to-treat population

	Females (*n* = 72)	Males (*n* = 230)
Any treatment-related adverse reaction*	27 (37^Ⴕ^)	67 (29^Ⴕ^)
Unspecified complaints about the mouth or jaw	15 (55^#^)	30 (45^#^)
Complaints about the teeth	5 (19^#^)	10 (15^#^)
Complaints about the temporomandibular joint	2 (7^#^)	15 (22^#^)
Complaints about the jaw muscles	2 (7^#^)	8 (12^#^)
Psychological complaints associated with the use of the appliance	0	2 (3^#^)
Headache or clenching	3 (11^#^)	2 (3^#^)

Data are the number (%) of participants reporting the event

*Rated by an investigator as probably or possibly related to the intervention

^Ⴕ^Based on the total numbers having one or several types of reactions

^#^Based on the numbers of a specified adverse reaction

## Discussion

That the population of OSA patients undergoing treatment is male dominated has been described previously [8, 19] and observed herein, where our study sample was 73% male. Females are reported to have received an OSA diagnosis when they are significantly older than males. Similar to the results herein, in their German population-based cohort Fietze et al [10] reported a mean age at diagnosis of 61 years for females, compared with 53 years for males. The subjects in our study were enrolled in a consecutive order when they fulfilled inclusion/exclusion criteria which reflects the higher proportion of males and it mirror the actual difference in age between gender in this OSA population.

In a cohort of more than 1,000 Spanish participants referred for polysomnography [7], the overall BMI was similar to our Swedish participants. However, Nigro et al [7] found a higher prevalence of morbid obesity (BMI ≥ 35 kg/m^2^) among females.

Our study hypothesis, that female participants with OSA respond better to OA treatment than males was not supported, in terms of both the proportion of responders and AHI decrease, either overall or within mild, moderate, or severe OSA severity groups. These results are inconsistent with those by Vecchierini et al [8], who concluded that overall treatment success and complete response rates were significantly higher in females than in males. These diverging results might be explained by study differences in follow-up times, which were 3–6 months in the Vecchierini et al study compared with one year herein. Different definitions of treatment response were also used. Vecchierini et al [8] defined a responder as “baseline AHI reduced by 50 percent,” whereas we considered a responder to have AHI < 10 at follow-up and/or ≥50% reduction from baseline. In addition, Vecchierini et al used a long titration process, whereas herein mandibular protrusion was predefined as 75% of the maximum protrusion capacity at the start of treatment. There were also differences in dropout rates. Vecchierini et al reported that 14% of females and 18% of males dropped out, whereas herein the rates were about double, 29% and 39%, respectively. The higher drop-out rate herein may have been due to our longer follow-up time and the fact that we collected an interim follow-up polygraphy after eight treatment weeks, at which time non-responders were excluded.

Nigro et al [7] reported that females with OSA are more likely to report tiredness, sleep onset insomnia, and morning headaches, and are less likely to complain of “typical” OSA symptoms. OA therapy reduces headache frequency [19], a positive effect that seems to improve with longer use [20]. Vecchierini et al [8] also found a significantly enhanced reduction in morning headaches among females compared with males in their short-term study. However, when headache outcome was defined as “once a week or more” in our one-year study, there was no gender difference. Besides the different study durations, the headache definition may explain the discrepant findings. However, despite these differences, both studies reported significantly reduced headache frequencies.

Improved daytime sleepiness has been reported with OA treatment, in the range of 1.7–6.6 points on the ESS [21–24]. Our sample showed improvements of 4.2 and 3.6 ESS points for females and males, respectively. This nonsignificant gender difference is consistent with results by Vecchierini et al [8]. Though the ESS has been validated for use with a range of sleep disorders patients, it is regarded as relatively insensitive in sleep apnea [17], and an ESS score > 10 is not significantly associated with AHI [10].

Participant perception of treatment effects was measured with the FOSQ, which assesses the impacts of disorders of excessive sleepiness on activities of daily living and quality of life. Herein, we found that positive changes among females and males did not differ significantly. Marklund et al [25] reported a mean FOSQ index reduction of 1.2 during their four-month study, very similar to the outcome herein of a mean improvement of 1.1 among both genders. However, this level of change is also similar to that achieved with placebo OAs [25].

Participants’ own ratings of their experiences with OA therapy using the PGIC were remarkably high. Both females and males rated their outcomes as “very much or much improved” in 94% of cases. This large improvement must be considered in relation to the fact that we only analyzed the per-protocol population, and thus excluded those who discontinued the study.

As with all treatment types, OA therapy is subject to certain adverse reactions. That we found a higher degree of treatment-related adverse reactions among females may mirror a real-world phenomenon, as adverse reaction reporting was made by all participants who started OA treatment (i.e., the intention-to-treat [ITT] population).

The study was not without some shortcomings. First, the larger study was not statistically powered to detect gender differences. However, the differences in proportions of responders were very small and absolute changes in AHI were almost equal. Second, analysis of the ITT population may have led to different results. The proportion of participants who withdrew was larger among males than females (39% and 29%, respectively). Finally, the degree of OA mandibular protrusion was significantly larger among males; indeed, the absolute range of mandibular motion is larger among males [13] and herein we predefined the same protrusion degree as 75% of the individual maximum capacity for all patients. This may have favored males who generally have a longer upper airway length and a higher propensity for collapsibility [26, 27].

## Conclusion

Both females and males with OSA respond well to OA therapy, with nonsignificant gender differences in study outcomes. Thus, according to these findings, the study hypothesis that females respond better than males to OA treatment is rejected.
